# Capacity Development through the US President’s Malaria Initiative–Supported Antimalarial Resistance Monitoring in Africa Network

**DOI:** 10.3201/eid2313.170366

**Published:** 2017-12

**Authors:** Eric S. Halsey, Meera Venkatesan, Mateusz M. Plucinski, Eldin Talundzic, Naomi W. Lucchi, Zhiyong Zhou, Celine I. Mandara, Hawela Moonga, Busiku Hamainza, Abdoul Habib Beavogui, Simon Kariuki, Aaron M. Samuels, Laura C. Steinhardt, Don P. Mathanga, Julie Gutman, Yves Eric Denon, Aline Uwimana, Ashenafi Assefa, Jimee Hwang, Ya Ping Shi, Pedro Rafael Dimbu, Ousmane Koita, Deus S. Ishengoma, Daouda Ndiaye, Venkatachalam Udhayakumar

**Affiliations:** Centers for Disease Control and Prevention, Atlanta, Georgia, USA (E.S. Halsey, M.M. Plucinski, E. Talundzic, N.W. Lucchi, Z. Zhou, A.M. Samuels, L.C. Steinhardt, J. Gutman, J. Hwang, Y.P. Shi, V. Udhayakumar);; US Agency for International Development, Washington, DC, USA (M. Venkatesan);; National Institute for Medical Research, Tanga, Tanzania (C.I. Mandara, D.S. Ishengoma);; National Malaria Control Centre, Lusaka, Zambia (H. Moonga, B. Hamainza);; Mafèrinyah Rural Health Research Center, Mafèrinyah, Guinea (A.H. Beavogui);; University Gamal Abdel Nasser of Conakry, Conakry, Guinea (A.H. Beavogui);; Kenya Medical Research Institute, Kisumu, Kenya (S. Kariuki);; US Centers for Disease Control and Prevention, Kisumu (A.M. Samuels);; University of Malawi College of Medicine, Blantyre, Malawi (D.P. Mathanga);; National Malaria Control Program, Cotonou, Benin (Y.E. Denon);; Rwanda Biomedical Center, Kigali, Rwanda (A. Uwimana);; Ethiopian Public Health Institute, Addis Ababa, Ethiopia (A. Assefa, P.R. Dimbu);; National Malaria Control Program, Luanda, Angola (P.R. Dimbu);; University of Bamako, Mali (O. Koita);; Université Cheikh Anta Diop de Dakar, Senegal (D. Ndiaye)

**Keywords:** malaria, antimalarial drugs, Africa, capacity building, artemisinins, antimicrobial resistance, genetic markers, tropical medicine, global health security, parasites

## Abstract

Antimalarial drug resistance is an evolving global health security threat to malaria control. Early detection of *Plasmodium falciparum* resistance through therapeutic efficacy studies and associated genetic analyses may facilitate timely implementation of intervention strategies. The US President’s Malaria Initiative–supported Antimalarial Resistance Monitoring in Africa Network has assisted numerous laboratories in partner countries in acquiring the knowledge and capability to independently monitor for molecular markers of antimalarial drug resistance.

“One finger does not kill a louse.” — Kikuyu proverb

Substantial recent progress has been made in malaria control, particularly in sub-Saharan Africa. During the past 5 years, malaria mortality rates have declined 31% in the region, an achievement attributable to many factors (*1*). From 2010 to 2015, the proportion of at-risk persons sleeping under an insecticide-treated net in Africa increased from 30% to 53%, the proportion of febrile children evaluated with a malaria diagnostic test at a public facility increased from 29% to 51%, and the proportion of pregnant women receiving 3 doses of preventive antimalarial treatment increased 5-fold. Another development is the growing availability of oral artemisinin-based combination therapies (ACTs), which rely on an artemisinin plus a longer-acting partner drug from another class to treat uncomplicated malaria (*2*). ACTs, which are quick-acting, inexpensive, and generally well tolerated, have been critical in treating millions of cases of uncomplicated malaria each year, reducing risk of progression to severe disease and death. The widespread availability and use of ACTs has been recognized as a main contributor to the decline in cases in Africa (*3*).

However, recent studies from Southeast Asia have identified the emergence and spread of *Plasmodium falciparum* parasites that are less susceptible to both artemisinin and the partner drug component of ACTs (*4*,*5*). International malaria control efforts experienced a similar setback in the 1950s, when chloroquine resistance first surfaced on the Thailand–Cambodia border and spread to Africa within 2 decades (*6*), and again in the 1970s with the rise and spread of *P. falciparum* parasite populations resistant to sulfadoxine/pyrimethamine (*7*). Fortunately, genetic markers of resistance to antimalarial drugs, including those for artemisinins (*8*) and partner drugs (*9*), are now used more easily to rapidly detect and track the spread of potentially resistant parasites.

The growing threat of antimalarial drug resistance is a major concern of the global health community. The US President’s Malaria Initiative (PMI), established in 2005 to support countries in scaling up malaria prevention and control efforts, now covers 19 countries in Africa plus the Greater Mekong subregion. PMI has helped provide nearly 400 million ACT treatment courses since its inception (*10*). During 2005–2015, the Global Fund to Fight AIDS, TB, and Malaria supplied an additional 582 million treatments (*11*). Losing these medications to drug resistance would not only jeopardize the progress achieved in reducing malaria infections in recent years but also threaten global health security by putting millions of additional persons at risk for death from malaria.

To monitor whether a country’s recommended ACTs remain efficacious, therapeutic efficacy studies (TESs) should be conducted at least every 2 years. TESs use a standard World Health Organization (WHO) protocol (*12*) to enroll uncomplicated malaria patients, who are given a quality-controlled ACT and monitored over 4–6 weeks for parasite clearance or the reappearance of parasites matching the infecting strain. Pending confirmation, therapeutic efficacy rates falling below 90% indicate an ACT may no longer be optimal for a given region or country. The US Agency for International Development’s support of a broad network of TES sites in the Greater Mekong subregion, begun in 2006, was instrumental in identifying suboptimal efficacy rates in the region. Subsequent PMI support for countries to conduct TESs in the Greater Mekong subregion and across sub-Saharan Africa continues to provide crucial data on the clinical efficacy of ACTs.

In addition to monitoring therapeutic outcomes in a TES, WHO also recommends testing the collected samples for genetic markers of antimalarial drug resistance to provide insight into the molecular underpinnings of treatment failure. Recently, WHO listed specific polymorphisms in the propeller domain of the *kelch 13* (*K13*) gene that could be used to identify suspected artemisinin-resistant parasites (*13*). Even though investigating for the presence of *K13* and other genetic mutations is an important complement to the primary outcome of TESs, it often falls outside a routine TES’s scope and budget and is often beyond a country’s capability.

To address this shortcoming, the PMI-supported Antimalarial Resistance Monitoring in Africa (PARMA) Network was created in 2015 with 2 primary objectives: 1) to assist PMI countries in testing samples from TESs for genetic markers associated with antimalarial drug resistance; and 2) to support training and capacity building of African collaborators who possess sufficient infrastructure in laboratory (e.g., real-time PCR, thermocyclers, gel electrophoresis) and bioinformatics (e.g., computer with sufficient memory and processing power) to incorporate these assays in future studies. By using dried blood spot samples collected during a standard TES, PMI’s support of additional testing through PARMA requires no extra blood draw or inconvenience to patients. After TES completion, samples are brought to the Centers for Disease Control and Prevention (CDC; Atlanta, GA, USA) Malaria Branch (Division of Parasitic Diseases and Malaria, Center for Global Health) by a laboratory-based trainee from the participating country partner. At CDC, trainees participate in 4–6 weeks of molecular laboratory training to process their countries’ recently collected TES samples, with the goal of generating antimalarial drug resistance marker data. Under the tutelage of CDC personnel, trainees learn molecular methods of testing for resistance markers to artemisinins and other drugs used in the treatment and prevention of malaria (Table). These methods may include DNA isolation, photo-induced electron transfer PCR (*14*), TaqMan-based real-time PCR (e.g., for *mdr1* gene copy number), Sanger sequencing, and PCR techniques that distinguish whether a recurrent infection matches the initial parasite (recrudescence) or is a new one (reinfection) (*15*). In addition to receiving instruction and training in the laboratory, trainees receive training in bioinformatics analysis and guidance on interpreting their findings, which they are encouraged to share with global monitoring entities such as WHO and the Worldwide Antimalarial Resistance Network. Depending on time, appropriateness, and interest level, other molecular (e.g., *hrp2* gene deletion associated with false-negative malaria rapid diagnostic test results) and nonmolecular (e.g., serology) training may be offered to strengthen laboratory capacity in support of malaria control and elimination programs. While at CDC, trainees interact with epidemiologists and public health professionals who provide advice on integrating the newly acquired knowledge and skills to benefit their country’s national malaria control program.

**Table:** Antimalarial drug resistance genes that may be analyzed by the President’s Malaria Initiative–supported Antimalarial Resistance Monitoring in Africa Network*

Gene	Chromosome	Type of mutation	Antimalarial drug(s) associated with resistance
*pfcrt*	7	Polymorphism	Amodiaquine, chloroquine
*pfmdr1*	5	Polymorphism	Amodiaquine, chloroquine, lumefantrine, quinine
		Change in copy number	Mefloquine
*pfdhfr*	4	Polymorphism	Pyrimethamine
*pfdhps*	8	Polymorphism	Sulfadoxine
Propeller domain of *kelch*	13	Polymorphism	Artemisinin derivatives (e.g., artesunate)
*plasmepsin 2* and *3*	14	Change in copy number	Piperaquine

*Markers are selected based on the needs and priorities of each country’s malaria control program. This list will be expanded as additional molecular markers are identified.

Much as in the medical adage “see one, do one, teach one,” PMI envisions its support of training at CDC as the first step in the knowledge transfer process. The PARMA Network’s experience with its first partner, the Department of Parasitology of Université Cheikh Anta Diop de Dakar in Senegal, illustrates how these training visits foster proficiency and self-sufficiency. After completing 6 weeks of molecular training at CDC in 2015, 2 trainees from Senegal returned to their laboratory and, a few months later, were visited by a CDC Malaria Branch representative who supported the successful implementation of molecular testing methods in the Université Cheikh Anta Diop de Dakar’s malaria laboratory. In early 2016, the Senegal laboratory used high-resolution melting and Sanger sequencing methods to determine the presence of antimalarial drug resistance molecular markers, including *K13*, on samples from TESs in their own country. Rounding out the process in August 2016, Université Cheikh Anta Diop de Dakar and CDC partnered in teaching a 1-week regional training session, Advanced Molecular Detection Tools and Analysis for Malaria, at the university. Laboratory workers from the PMI countries Mali, Senegal, and Zimbabwe and the non-PMI country Morocco attended using their own funding.

Following on Senegal’s success, additional PMI countries are now participating in the PARMA Network and have sent samples, trainees, or both to CDC (Figure). Countries already possessing results include Angola, Guinea, Kenya, Malawi, Mali, Tanzania, and Zambia. Other countries, including Benin, Ethiopia, and Rwanda, will participate in the network with their next TES. The ultimate goal is to enable institutions in Africa to independently offer molecular monitoring, diagnostic training, and associated support to other countries. Building capacity through this type of regional partnership will harmonize testing and quality assurance protocols among African countries. A sustainable model allows countries to enhance ongoing TESs by independently identifying antimalarial drug resistance markers in the local parasite population. Furthermore, with its current 34 sites spanning 11 countries, the expanding PARMA network provides an opportunity for investigators to collaborate in analyzing trends in malaria data over time and space. Capabilities acquired, connections forged, and lessons learned through this laboratory network can be used to combat other infections of importance to global health security, ranging from viral hemorrhagic fever to pandemic influenza to emerging arboviral disease.

**Figure:**
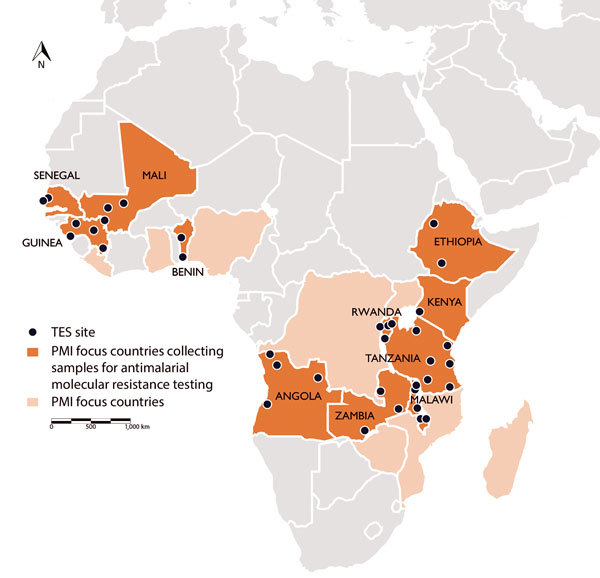
US President’s Malaria Initiative (PMI)–supported therapeutic efficacy study (TES) sites, where samples were collected to test for molecular markers of antimalarial drug resistance, 2015–2017.

Improving molecular surveillance capability in Africa could preserve antimalarial drug efficacy on the continent. Molecular surveillance can complement conventional TES methods and serve as an early warning system to trigger and direct follow-up investigations in areas of suspected resistance. Accelerating the confirmation of resistance and assisting countries in identifying appropriate actions are consistent with the aim to prevent, detect, and respond to human disease threats in the name of global health security. Whether that entails targeted interventions (e.g., heightened case surveillance, intensive indoor residual spraying of insecticide); switching to 1 of the other 5 WHO-approved ACTs; or developing a new option remains to be determined. Several innovative compounds show promise (*16*), including those possessing substantial differences from existing antimalarial drugs in their class and those with completely novel mechanisms of action; ongoing studies continue to produce safety and efficacy data. Available strategies include adding a third drug to the current 2-drug approach of treating uncomplicated malaria (triple therapy) (*17*), exploring the safety and efficacy of sequential administration of different ACTs, and extending the treatment duration of existing therapies (*18*). Regardless of the strategy chosen, surveillance programs such as PARMA and the PMI-supported TES network will be instrumental in keeping malaria control in Africa a step ahead of the parasite.
